# Integrated Sensor-Composite Material Platform for High-Resolution Voltage Mapping in Tissue-Mimicking Models

**DOI:** 10.1021/acsomega.5c12602

**Published:** 2026-03-16

**Authors:** Kajal C. Jain, Richa Srivastava, Armin Jamali, Frank Goldschmidtboeing, Peter Woias, Laura M. Comella

**Affiliations:** † Laboratory for Design of Microsystems, Department of Microsystems Engineering - IMTEK, University of Freiburg, Freiburg Im Breisgau 79110, Germany; ‡ Cluster of Excellence LivMatS, FIT - Freiburg Center for Interactive Materials and Bioinspired Technologies, University of Freiburg, Freiburg Im Breisgau 79110, Germany; § Institute of Energy Efficient Mobility, 38992 Karlsruhe University of Applied Sciences, Karlsruhe 76133, Germany

## Abstract

Accurate mapping of voltage distributions in tissue-mimicking materials (TMMs) is essential for the reliable design and validation of electrical stimulation therapies. Conventional phantoms with embedded commercial electrodes often suffer from limited spatial resolution and field mapping artifacts due to the electrode size and supporting structures. Here, we present a scalable sensor platform featuring custom copper sensor arrays (1.6 mm diameter, 1 cm spacing), each individually encapsulated by a dielectric layer and embedded in conductive PDMS/MWCNT composites (conductivity ∼0.24 S/m). This platform addresses a key limitation of existing embedded electrode approaches by improving spatial resolution and mapping accuracy while maintaining precisely known sensor coordinates and flexible placement within a conductive TMM. The system incorporates a robust, multiplexed electronic interface for automated, high-density voltage mapping. Voltage mapping experiments under identical AC stimulation performed at 100 Hz with measured signal amplitudes of 0.7 and 1 Vpp demonstrate that the sensor insulation technique enables high-resolution, symmetric voltage maps across the TMM with minimal measurement artifacts or distortion. This platform provides accurate visualization of voltage distributions, from which local electric fields can be inferred, and supports the rigorous preclinical development, validation, and calibration of advanced electrical stimulation protocols across diverse phantom geometries.

## Graphical abstract

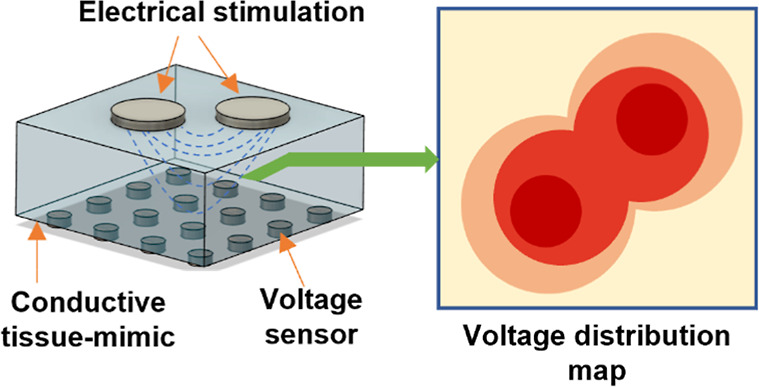

## 1 Introduction

Electrical stimulation of biological tissues is increasingly applied in biomedical research, therapeutic interventions, and implantable medical devices.^1–5^ Applications span from neural modulation and pain management to muscle rehabilitation and recovery, where the ability to deliver controlled electrical fields to well-defined regions is critical for efficacy and safety.^2,6,7^ A central challenge across these modalities is understanding and optimizing the spatial distribution of electric fields within complex biological environments, since unintended stimulation of adjacent tissues or nerves can lead to undesirable side effects and reduced therapeutic benefit.^8,9^ Reliable mapping of local voltage distributions in tissue-mimicking environments therefore represents a key enabling step toward precision electrical stimulation protocols.^9^

Transcranial electrical stimulation (tES) offers a representative case in which this challenge is particularly acute. As a noninvasive neuromodulation technique, tES delivers weak currents to the brain through scalp-mounted electrodes in order to influence neuronal activity in neurological and psychiatric disorders. The main modalities include transcranial direct current stimulation (tDCS), alternating current stimulation (tACS), and random noise stimulation (tRNS).^10,11^ The efficacy of these modalities depends on generating precise and localized electric fields at targeted brain regions, which are strongly influenced by stimulation parameters and the conductive properties of intervening tissues.^12,13^ As such, mapping voltage distributions within physical head phantoms has become an essential preclinical strategy for validating computational models, guiding electrode placement, and ensuring safe and effective protocols.^14–17^

Conventionally, phantoms for tES are fabricated from electrically conductive tissue-mimicking materials (TMMs) such as saline solution or saline-doped gels such as agar or gelatin. These platforms allow local voltage measurements through embedded electrodes and have supported early efforts in validating stimulation models.^15,16,18^ However, limitations arise primarily from the commercial measurement electrodes or sensors used in such systems. Sensors such as gold cup electrodes (10–20 mm diameter) are too large to be densely integrated into agar gel without significantly disturbing the local electric fields.^16^ Although smaller Ag/AgCl pellet electrodes (1–2 mm diameter) offer improved resolution,^15^ stabilizing them within conventional TMMs often requires dense support structures, which themselves distort the fields. Consequently, these constraints hinder accurate mapping of localized field variations, a requirement for focal applications such as tES where avoiding off-target stimulation is critical.^11,12^

To address this limitation, we previously developed a state-of-the-art material-driven approach by embedding custom copper sensor arrays directly into conductive polymer composites composed of polydimethylsiloxane (PDMS) doped with multiwalled carbon nanotubes (MWCNTs).^19,20^ This approach enables a seamless integration of finely patterned sensor networks, enabling precise geometric control, enhanced spatial resolution, and preserved electrical stability within the phantom. Importantly, this strategy shifts the focus from adapting phantoms to accommodate standard sensors, toward the codesign of the sensor–material interface as a unified, high-performance platform.

In the present work, we advance our sensor–material integration approach by introducing point electrodes with an electrical insulation of their connecting copper traces. These are embedded in a TMM made from a specially designed PDMS/MWCNT composite in combination with a mechanically robust interface layer for reliable signal acquisition. The novelty of this sensor design lies in its ability to achieve improved spatial resolution and mapping accuracy while preserving the inherent advantages of the previous material-driven approach, including flexible sensor placement and precisely known sensor coordinates.

Additionally, we design and implement an electronic hardware framework that enables automated, high-throughput measurements, further increasing the measurement accuracy and reproducibility. To benchmark these improvements, we directly compare our new insulated and automated electrode platform with our earlier noninsulated, manually operated system, which was limited by electrical cross-talk and reduced spatial accuracy.^19,20^ Through comparative analysis of voltage distributions within the TMM under constant AC stimulation, we investigate how the combination of sensors with insulated traces, a robust measurement interface, and an automated signal acquisition minimizes voltage-mapping artifacts associated with field distortions and enhances spatial mapping fidelity. These developments establish a scalable platform for phantom studies that supports preclinical research and lays the groundwork for improved design, validation, and calibration of electrical stimulation protocols for biological tissues.

## 2 Results and Discussion

### 2.1 TMMs for Sensor–Material Integration

Reliable voltage mapping requires embedding copper sensors in TMMs with stable and well-defined electrical properties. For both noninsulated and insulated sensor platforms, TMMs were synthesized from a single batch of PDMS doped with MWCNTs, exhibiting a frequency-independent conductivity of 0.24 S/m between 4 Hz and 1 kHz (see Methods). Sensors were integrated into these TMM slabs, each measuring 7 × 7 cm laterally and 5 mm thick, allowing for a direct comparison of performance in all subsequent voltage mapping experiments.

### 2.2 Noninsulated Sensor Platform

The noninsulated platform was fabricated by a direct embedding of copper sensors into the TMM. Such a copper sensor is composed of the actual measurement node, a solder pad for electrical connection to the outer world, and a copper track between both. In a so-called noninsulated approach, this whole sensor is made in one process step from a thin copper layer and is embedded into the TMM without any electrical isolation to the same. This means that the measurement node is not only in electrical contact with the TMM but also the tracks and solder pads (see Figure 1). This configuration serves as a baseline for evaluating crosstalk and local field distortion that can arise when multiple sensors are placed in close proximity without insulation.^19^ As such, the noninsulated copper sensor provides an essential reference for assessment of the effectiveness of sensor insulation in improving voltage mapping accuracy and spatial resolution described in Section 2.5.

**Figure 1:**
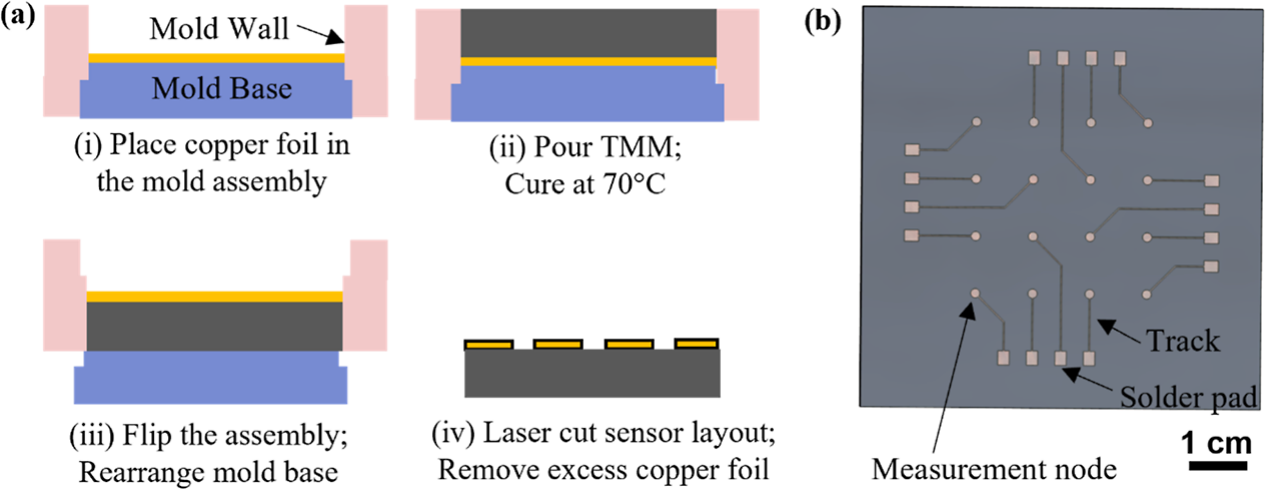
(a) Fabrication steps for embedded copper sensors into a TMM matrix. (b) Illustration of a TMM with embedded noninsulated copper sensors; each sensor consists of a measurement node, a connecting track, and a solder pad for electrical interfacing.

For fabrication, a separable PVC mold base and walls were assembled to create a cavity with the intended TMM dimensions of 7 × 7 cm × 5 mm. A 35 μm-thick copper foil sheet was placed on the mold base, allowing for subsequent integration of sensor structures (see Figure 1a, step (i)). The PDMS/MWCNT composite was then poured into the mold cavity, and the assembly was cured at 70 °C for 2 h to achieve a complete cross-linking and mechanical stabilization (step (ii)). The tight-fitting mold ensured precise control over the final slab thickness during pouring and curing.

Following curing, the copper foil was exposed, and the edges of the mold wall were aligned to the pilot laser of a precision laser cutting device (step (iii)). The desired sensor layout was defined by computer-controlled laser ablation (step (iv)).^19^ This process enabled a reproducible, high-resolution patterning of the copper sensors directly within the TMM, with a total cutting duration of approximately 30 min. After laser ablation, connecting wires were soldered onto the exposed solder pads to complete the electrical interface, shown in Supporting Figure S1. The number, spacing, and geometry of sensors can be readily adapted in this platform to support high-density mapping or custom layouts.

However, sensors embedded using this approach are prone to delamination due to mechanical stress, especially during the soldering of connecting wires and throughout subsequent voltage mapping measurements involving repeated handling. Although the connecting wires were fixated to the TMM using silicone to minimize mechanical stress at the solder pads, the sensors remained susceptible to delamination during extended or repeated use. These limitations highlight the need for a more robust and mechanically stable electronic interface to preserve sensor integrity and ensure a consistent performance during extended use. Therefore, a stabilized interface for signal acquisition was developed, as described in Section 2.4.

### 2.3 Insulated Sensor Platform

The insulated sensor platform featured a dielectric coating applied to all copper sensor tracks and solder pads, leaving only the measurement nodes in electrical connection with the TMM. This approach provided electrical isolation from both the TMM matrix and adjacent sensors while maintaining the design flexibility of our previous platform in Section 2.2. The insulation region was designed to have a defined width and thickness of 1 mm around the 0.3 mm tracks to ensure a robust isolation and reliable performance.

#### 2.3.1 Insulation Fabrication and Integration into TMM

The fabrication of the insulated sensor platform, outlined in Figure 2a, required a carefully orchestrated, multistep process that leveraged both precise mold design and manual control for a reliable sensor integration and robust insulation geometry. The mold wall features profiles of 5 mm for the main TMM body and 1 mm, realized as two sequential 0.5 mm layers, for the sensor insulation layer (Figure 2b; see also Supporting Information Figure S2). The process began with the placement of a 35 μm-thick copper foil into a custom-designed 3D-printed resin mold with a separable base and wall (step (i)). Electrically insulating PDMS was poured onto the copper foil and evenly distributed by doctor blading to form a uniform 0.5 mm-thick layer, as defined by the mold wall edges (step (ii)).

**Figure 2:**
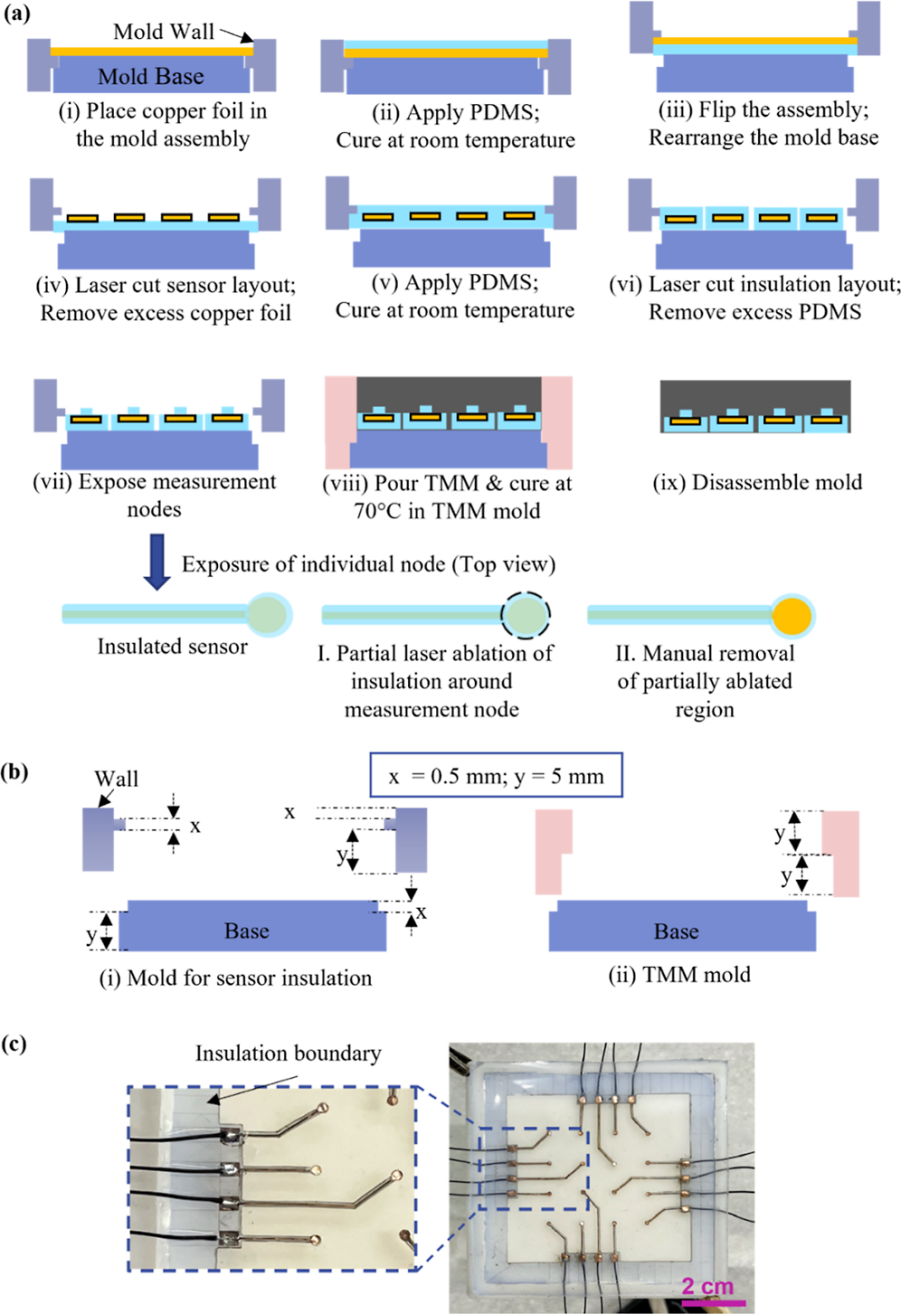
(a) Schematic cross-section illustration of the fabrication process for insulated sensor arrays embedded in TMM; (b) mold design for the realization of different thicknesses: “*x*” = 0.5 mm for the insulation layer, for a total insulation thickness of 1 mm, and “*y*” = 5 mm for the TMM; (c) photographs of an insulated sensor array embedded into clear PDMS for visual clarity, with 0.5 mm thick insulation boundary around the sensors, formed as a result of insulation process step (ii).

After curing, the assembly was flipped, and the base detached and repositioned within the mold to expose the uncoated copper surface to air (step (iii)). The sensor layout was then patterned onto the copper foil using laser ablation, during which a grid pattern was introduced in the surrounding copper regions to facilitate manual removal of excess foil (see Supporting Information Figure S6 “Sensor layout with grid lines”). The ablated grid sections were subsequently removed using tweezers, leaving the laser-defined sensor geometry intact (step (iv)). While the grid pattern may be adjusted, reproducible fabrication with a 100% yield is maintained (as observed in 5 samples) provided that the foil removal process minimizes tensile loading on the PDMS surrounding the sensor structures. Subsequently, a second PDMS layer of 0.5 mm thickness was applied by doctor blading (step (v)), resulting in an intended insulation region of 1 mm total thickness around the copper sensors.

An insulation width of 1 mm was defined in the CAD sketch (see Figure S3) and employed during the laser ablation process (step vi) to precisely pattern the insulation layout around the copper tracks and solder pads. This design choice, based on the laser tolerances, effectively maintained a safe margin around the 0.3 mm-wide sensor tracks, ensuring complete encapsulation and reliable electrical isolation. The selected width of 1 mm further proved adequate in compensating for minor alignment offsets encountered during fabrication, such as those arising from the laser pilot beam width of ∼35 μm and occasional human or focus-setting deviations when aligning the molds under the laser. Postfabrication measurement with vernier calipers revealed an average track width of 0.80 ± 0.05 mm among the 16 insulated copper sensors.

Exposing the circular measurement nodes (step vii) was achieved through a two-step approach: partial laser ablation at the node locations, followed by careful manual removal of residual PDMS with a scalpel. This approach minimized the risk of track damage by a laser and ensured access only to the intended regions required for electrical contact, maintaining a robust sensor alignment.

The insulated copper sensors were then transferred into a new TMM mold, with the exposed measurement nodes oriented upward. At this stage, the measurement nodes on the opposite side remained unexposed, ensuring that the sensors stayed securely anchored and did not shift or float during subsequent steps. The PDMS/MWCNT mixture was then poured and cured around the embedded structure (step (viii)), resulting in integrated composite devices suitable for voltage mapping experiments. After TMM formation (step ix), the solder pads can be accessed for wiring by manually exposing them with a scalpel.

Any remaining unexposed measurement nodes on the reverse side can similarly be revealed as needed by partial laser ablation, followed by manual removal. This approach also allows those nodes to be brought into contact with an additional TMM layer in multilayer structure fabrication, facilitating future interlayer mapping or connectivity. Direct visual inspection of sensor integration within a transparent PDMS matrix (Figure 2c) revealed that while the insulation was not perfectly symmetric around the sensor tracks, complete encapsulation of each track was consistently achieved. The influence of insulation asymmetry on the sensor platform performance remains to be systematically investigated for the future.

### 2.3.2 Structural Integrity and Thickness Evaluation

To further assess the quality of the encapsulation, the structural integrity was evaluated in a second sample. Individual—and totally insulated—sensors were immersed in a saline bath with an electrical conductivity of 1.07 S/m, with care taken to ensure all measurement nodes and tracks remained completely encapsulated by the dielectric layer, and the solder pads with connecting wire, being not fully insulated, were left outside the bath. Under these conditions, a constant current of 1 mA was applied at 1 kHz and the voltage was monitored at the immersed sensor through the connecting wire. No conductive pathway was detected, confirming that the dielectric barrier provided effective electrical isolation and prevents any direct electrical contact between the copper and the solution. However, as expected, residual capacitive coupling with amplitudes between 0 V and −20 mV was observed between the copper sensor and the conductive medium, with the insulation layer acting as a dielectric. The setup and results for the saline bath test are presented in Supporting Information Figure S4.

The insulation process was designed to yield a total thickness of 1 mm around the copper sensor tracks, comprising 0.5 mm on both the top and the bottom of the sensors. To assess the actual process outcome, the insulation boundary thickness (see Figure 2c), representative of the top insulation layer, was first measured by calipers, giving a value of approximately 0.52 mm. While this matched the intended design, caliper measurements for thin, soft materials like PDMS could be unreliable: excessive force on the calipers may compress or damage the material and fine tracks, while insufficient force could result in overestimated values. Caliper measurements of the mold edge profiles (Figure 2b) matched the nominal values, thereby ruling out dimensional inaccuracies due to mold fabrication as a source of error.

To complement the mechanical measurements, nondestructive optical profilometry was used to determine the local insulation thickness with high spatial resolution. This method analyzes reflected light and focus variation to generate high-resolution *z*-axis data along a scan line between two focal points—specifically, from the base plate (the substrate on which the insulated sensors were placed) up to the surface of the insulated track. This approach provided a detailed profiling of the dielectric layer, revealing thickness values ranging from 773.65 to 808.07 μm, which were close to the 1 mm design target. Some measurement error may result from the transparent nature of PDMS, which can cause internal refraction and interference from reflections of the embedded copper tracks (see Supporting Figure S5).

While the present technique offers a robust encapsulation, future improvements may focus on automated methods such as spin coating. Such refinements could potentially yield insulation layers down to tens of micrometers with tight tolerances, further reducing the electric field perturbations. However, as the insulation layer becomes thinner, increased capacitive coupling may occur in electrical measurements, while thicker insulation, though reducing capacitance, might lead to higher field disturbances. Alternatively, the capacitive artifacts can also be reduced by decreasing the thickness of the copper foil itself down to 12 μm, thereby increasing the effective thickness of the dielectric layer between the sensors and the external environment while maintaining the overall insulation thickness at 1 mm.

To fully understand the trade-offs, simulations should be performed in the future to investigate the effect of insulation thickness on electric field distribution and to determine the optimal insulation layer for a given application. Notably, the simple geometry of the present sensor platform makes it especially well-suited for a direct quantitative comparison with simulations as it reduces geometrical complexity and interpretation ambiguities. Ultimately, optimization of the insulation geometrical parameters will require a careful balance between geometric uniformity, capacitive effects, and impact on the electric field distribution to meet the desired performance criteria for different experimental and clinical scenarios.

### 2.4 Stable Sensor Interface for Voltage Distribution Measurements

To enable reliable and reproducible measurements of the voltage distributions across the sensor arrays, we developed a robust modular interface and a signal processing system (Figure 3). The sensor interface board features a centrally positioned cavity to accommodate the TMM, ensuring low-resistance, robust connections from all sensing nodes via presoldered wires. For applications in multilayer TMMs, the design enables stacking of multiple boards to interface with sensors from each layer.

**Figure 3:**
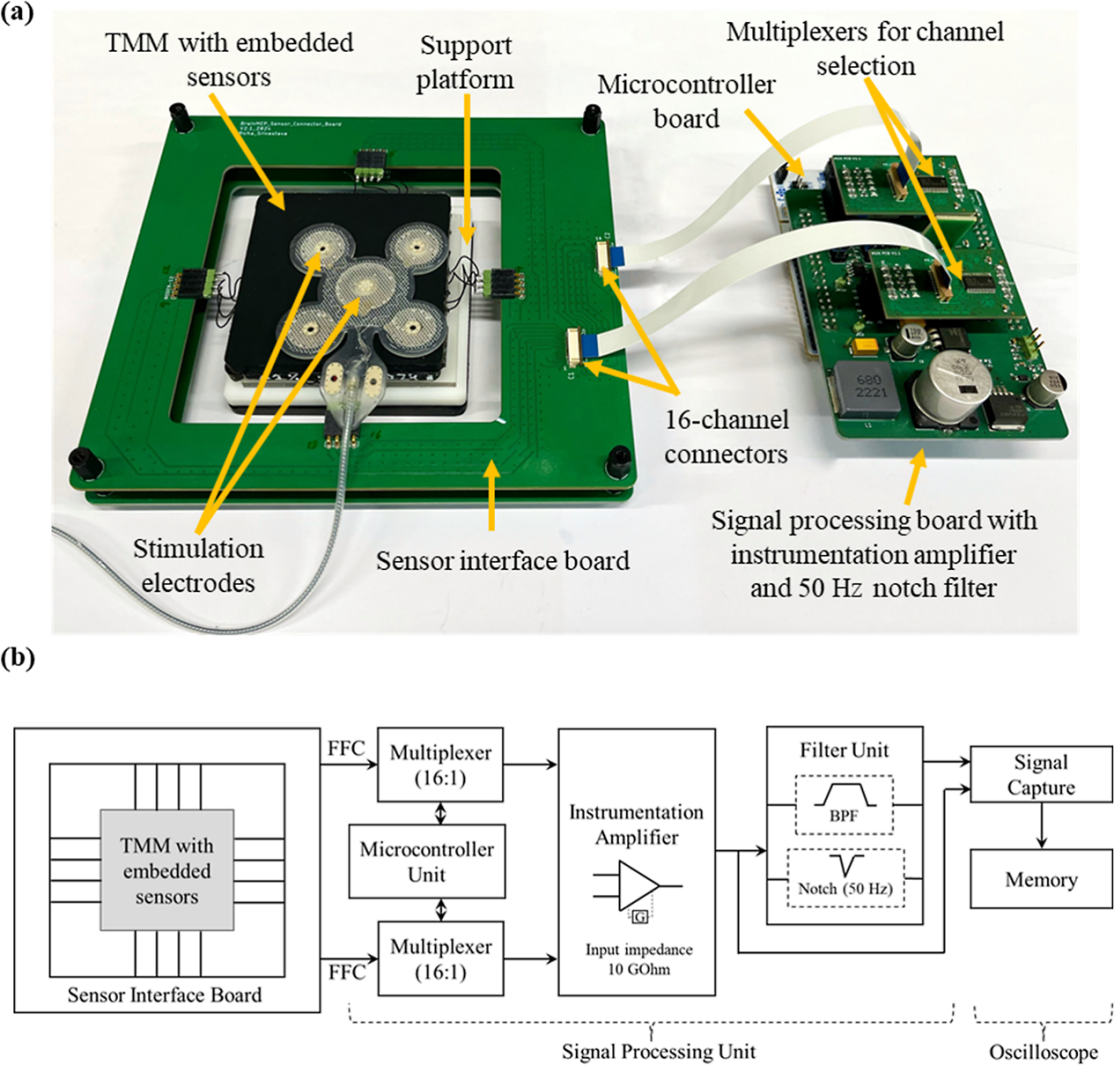
(a) Measurement interface for voltage mapping in tissue-mimicking materials (TMMs). Labeled components include: stimulation electrodes, TMM with embedded sensors, support platform for phantom placement, sensor interface board, two 16-channel connectors linked to dual 16-channel multiplexers for channel selection via FFC cables, microcontroller board for automated control, and the signal processing board integrating an instrumentation amplifier and a 50 Hz notch filter. The use of two multiplexers and two 16-channel connectors enables differential voltage measurements between any selected pair of sensor nodes, with the selected channels routed to the input terminals of the amplifier. (b) Block diagram of the voltage measurement from the embedded sensors (insulated and noninsulated) in TMMs.

A key feature of the platform is the use of dual 16-channel connectors, each paired with a multiplexer, allowing all sensors in the array to be simultaneously connected. This setup enables a flexible, automated selection of any two sensor nodes as differential inputs to the instrumentation amplifier. As a result, diverse measurement protocols are supported, including a selection of electrode pairs from the same or different TMM layers in a multilayer phantom. In this work, the limit of detection (±18 V) and the sensitivity (0.28 μV_(p‑p)_/20 mV) of the sensor arrays are determined by the instrumentation amplifier in the measurement hardware (AD620A).^21^

Electrical stimulation was delivered through one channel of a four-channel EASEE neurostimulation electrode pair (10 mm Pt/Ir anode, 15 mm Pt/Ir cathode), supplying a constant 14 μA sinusoidal current at 100 Hz for both the noninsulated and insulated sensor-integrated platforms. During operation, the combined signal processing and microcontroller unit streamlined data collection and contributed to noise minimization. The integrated multiplexer allowed for rapid, automated cycling through up to 16 sensor nodes under precise microcontroller control. An instrumentation amplifier provided substantial common-mode noise rejection, while its high input impedance of 10 GΩ ensured that a negligible stimulation current was diverted into the measurement circuitry. To further improve the signal quality, a 50 Hz notch filter was applied, which effectively eliminated interference from line frequency noise. After signal conditioning, voltage signals obtained from the sensor nodes were recorded and visualized by using an oscilloscope. Additionally, a 14 bit analog-to-digital converter (ADC) is integrated into the signal processing board to facilitate digital signal acquisition. Looking forward, we plan to utilize the onboard microcontroller to acquire sensor data directly and display results on a custom software interface, enabling automated data logging, real-time analysis, and enhanced accessibility for users.

Conductive electrode paste was applied to establish contact between the stimulation electrodes and the TMM surface; however, the current magnitude was limited by the relatively high contact impedance of 5.7 MΩ (measured between the anode and cathode) at this interface. In the absence of conductive paste, no measurable current flowed between the stimulation electrodes as the contact resistance at the electrode–TMM interface was effectively infinite. For future optimization, reducing the contact resistance between electrodes and the TMM should be prioritized to improve system performance at higher stimulation currents. Potential strategies include roughening or otherwise modifying the TMM surface to increase the effective contact area. In addition, employing a constant current source with a higher compliance voltage may further mitigate the effects of residual contact resistance. These approaches, combined with the present stable and semiautomated signal acquisition platform, will further enhance measurement reliability in future electrical stimulation studies. Full technical details and wiring protocols are provided in the Methods chapter.

### 2.5 Voltage Mapping within Tissue-Mimicking Materials

Using the measurement platform described above, we evaluated the effect of sensor insulation by recording voltage distributions in both noninsulated and insulated sensor platforms under identical stimulation protocols. Using the differential measurement setup, voltages were systematically mapped across the embedded copper sensors, while a constant alternating current of 14 μA was applied at 100 Hz. Differential voltages were recorded across 15 copper sensors with respect to a common reference sensor, as shown in Figure 4a. The resulting root-mean-square voltage distributions for both the insulated and noninsulated sensor platforms are plotted in Figure 4b.

**Figure 4:**
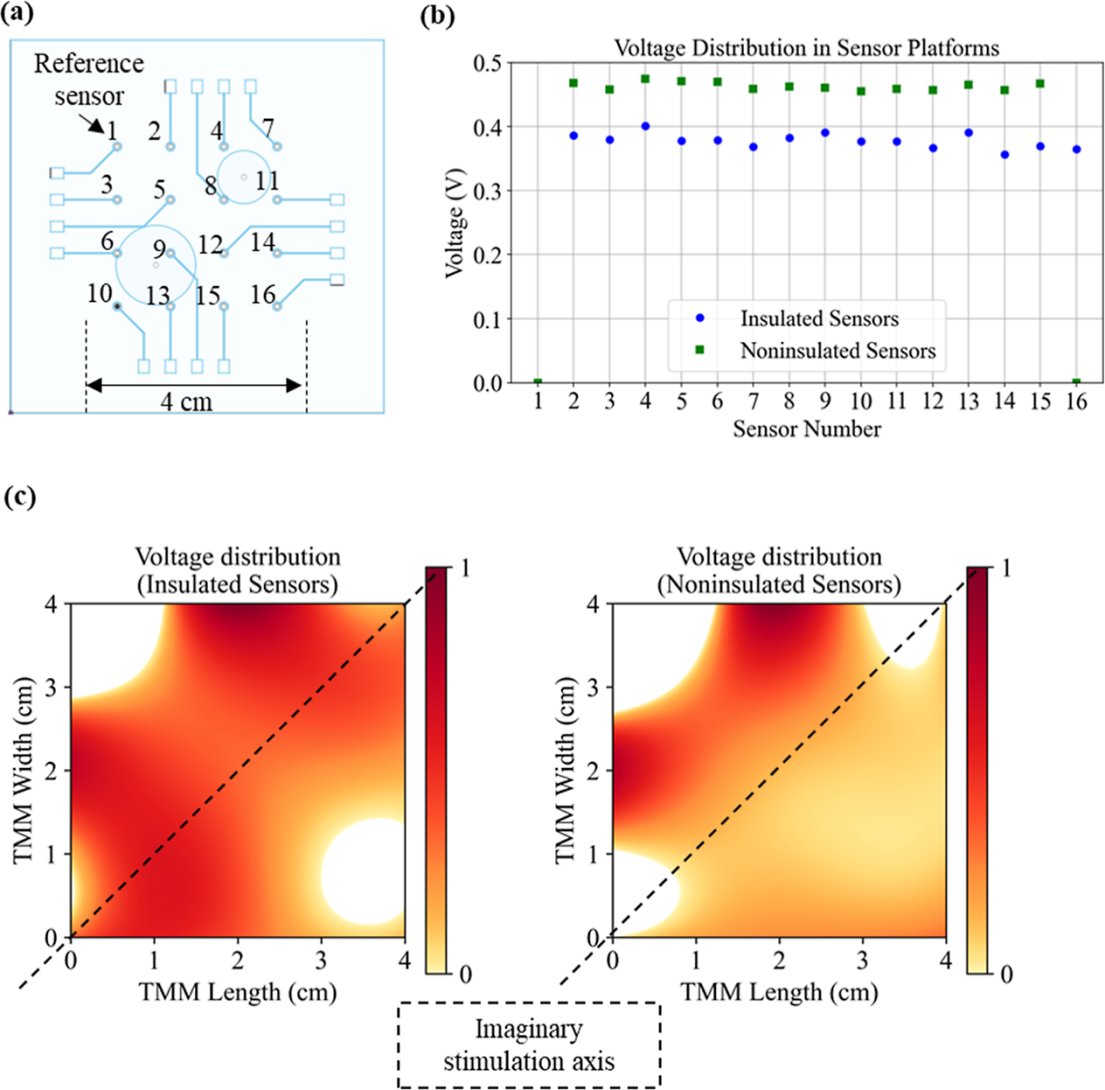
Comparative voltage mapping in TMM phantoms with noninsulated and insulated sensor platforms. (a) Schematic showing the positions of the embedded sensor nodes with stimulation electrode locations marked. The smaller and larger circular represent the anode and the cathode, respectively. (b) Differential root-mean-square (RMS) voltage distributions for both platforms under identical AC stimulation (14 μA, 100 Hz, 1.25 Vpp), illustrating a lower overall voltage drop in the noninsulated TMM and sharper spatial features in the insulated array. (c) Heatmaps of normalized RMS voltage distributions across the TMM surface, generated by cubic interpolation. The dashed line indicates the imaginary diagonal axis through the stimulation electrodes. The insulated platform shows near-symmetric voltage distribution, while the noninsulated platform displays clear asymmetry and field concentration due to current spreading.

Notably, the voltages measured with the insulated sensor platform were consistently lower than those from the noninsulated sensor platform. Despite this difference in magnitude, the spatial voltage distributions across the two platforms showed near-identical agreement, with a Pearson correlation coefficient of 0.99 after excluding one delaminated sensor in the noninsulated platform (sensor 16). This observation indicates that the voltage drop across the TMM in a noninsulated platform is lower compared to that of the insulated platform, given that a constant stimulation current was maintained. This trend can be explained by the difference in effective conductive path length: the extended length of exposed copper in the noninsulated sensor platform provides shorter, lower-resistance pathways for current to flow within the TMM. Our frequency-sweep impedance measurements showed that the TMM exhibited stable, frequency-independent conductivity over the tested range of 4 Hz to 1 kHz, indicating predominantly resistive behavior under these experimental conditions. Therefore, as expected for a resistive material, a lower-resistance pathway produced a reduced voltage drop at a constant current.

It should be noted that while capacitive coupling due to the insulation layer is physically possible and has been observed as a small artifact (with amplitudes between 0 and −20 mV at 1 kHz), the observed voltage signal amplitudes during mapping experiments were much larger (0.7–1.5 Vpp) and at a lower frequency of 100 Hz. Impedance analysis of the insulated sensor platform further confirmed that, at 100 Hz, the phase angle remains close to 0°, indicating predominantly resistive behavior and negligible capacitive contribution under the experimental conditions (see Supporting Information Figure S7). Thus, under our measurement conditions, capacitive artifacts are considered negligible and are unlikely to account for the observed difference in voltage drop between platforms. Notably, individual sensors exhibit different apparent cutoff frequencies at higher frequencies (>10 kHz), indicating a sensor-dependent frequency response of the TMM–sensor system. A detailed investigation of these high-frequency effects, including their dependence on sensor geometry and position, is beyond the scope of the present study and will be addressed in future work.

As shown in the voltage plots in Figure 4b, pronounced peaks are evident at sensors 4 and 13, corresponding to regions directly beneath the stimulation electrodes. In the insulated sensor platform, an additional peak is observed at sensor 9—also located under stimulation electrodes—which is absent in the noninsulated platform. In the noninsulated configuration, the voltage profile beneath the electrodes appears smoother, likely due to a diversion of current into the noninsulated track of sensor 5, located directly beneath a stimulation electrode near sensor 9. Moreover, the voltage differences between adjacent sensors near these peak positions are more pronounced in the insulated array, highlighting an increased spatial contrast, whereas in the noninsulated array, voltage transitions are smoother across this region. These observations suggest that the insulated platform provides enhanced spatial resolution in voltage measurements, better capturing local electric field gradients that are otherwise masked by current spreading in noninsulated designs.

Normalized RMS voltage heatmaps for both sensor platforms were generated by interpolating the discrete measurement data onto a fine spatial grid using cubic interpolation, enabling visualization of the voltage distribution across the TMM surface (see Figure 4c). Sensor 1 was used as the reference for normalization, and the minimum–maximum normalized voltage values corresponding to 0 and 1 are 0.357 vs 0.391 V for the insulated sensor platform and 0.455 vs 0.471 V for the noninsulated platform. Cubic interpolation was chosen to improve visual clarity and to represent smooth spatial transitions between neighboring discrete measurement points while preserving the underlying qualitative voltage distribution trends. The normalized RMS voltages without interpolation are provided in the Supporting Information Figure S8. To further analyze the spatial pattern of the mapped voltage field, we considered an imaginary diagonal axis running from the bottom left corner to the top right corner of the TMM, passing through the centers of both stimulation electrodes. Along this axis, a symmetric voltage distribution would be anticipated in the absence of field distortion, as seen in the simple FEM simulation model of the TMM (Supporting Information, Figure S9). The simulation predicts the highest field strength directly beneath the stimulation electrodes, with a gradual decrease in field strength away from the electrodes, producing a smooth, symmetric voltage gradient along the stimulation axis. This expected symmetry was readily observed in the insulated sensor platform, where the voltage map appeared largely balanced across the stimulation axis. Notably, while the heatmap shows a slight shift in the axis of symmetry, this can be attributed to interpolation-induced bias.

In contrast, the noninsulated platform exhibited a marked asymmetry, with the field clearly more concentrated toward one side of the TMM. Our prior work also demonstrated that extended and exposed sensor lengths can create preferential current pathways and disrupt voltage distribution symmetry.^19^ These results reinforce that the use of insulated sensors significantly reduces field distortion and yields a voltage distribution that more faithfully represents the intended experimental design.

### 2.6 Stability and Reproducibility of the Sensor Platforms

The system is highly stable and deterministic: the TMM conductivity is consistent, and the stimulation electrode and sensor geometry are fixed. The sinusoidal voltages recorded at the sensors remained stable for the duration of the experiment (∼15 min), with a maximum observed random variation of few μV to 10 mV in the sinusoidal peaks. This was verified by measuring the voltage at an arbitrarily chosen sensor, once at the beginning and at the end of the measurement. A raw voltage signal applied to the TMM and the differential voltage recorded between two sensors is shown in Supporting Information Figure S10.

In prior work, a similar TMM composition sandwiched between copper foils maintained stable conductivity between 4 Hz and 1 kHz, even after 72 days.^22^ Additionally, independent voltage mapping measurements performed on a separately fabricated TMM with a lower conductivity (0.16 S/m) produced voltage distributions that closely matched those obtained from the TMM platforms in this work, with a Pearson correlation coefficient of 0.95 when excluding a single sensor with poor contact (Supporting Information Figure S11).

Copper is known to be susceptible to oxidation over longer periods, depending on environmental conditions. Encapsulation of the copper sensors within the PDMS, in the case of insulated sensors, substantially limits direct electrochemical interaction with the surrounding conductive media. PDMS-based coatings have been shown to act as effective diffusion barriers that suppress copper oxidation and corrosion by reducing ionic transport and surface reactivity, even in electrically conductive environments.^23,24^ Additionally, our fabrication process is compatible with any laser-cuttable metal foil, allowing the use of more oxidation-resistant materials such as gold, platinum, and stainless steel to further prolong the shelf life of the TMM platform.

### 2.7 Comparison with State-of-the-Art Voltage Mapping Techniques

Previous strategies for mapping voltage distributions in anatomical phantoms have typically relied on probe- or needle-based measurements inserted at specific locations.^14,25,26^ While these approaches can achieve high local spatial resolution, the measurement coordinates are difficult to reproduce accurately across experiments, and repeated probe insertion may cause material damage or locally alter conductivity.

The insulated sensor platform developed in this work consists of millimeter-scale copper point electrodes (1.6 mm diameter) arranged with a 1 cm sensor pitch with 16 sensor nodes embedded over a 4 × 4 cm TMM area at fixed coordinates. By embedding individually insulated sensors with an effective spatial resolution of 1 cm directly within the TMM, and providing a mechanically robust interface, this platform allows high-density, reproducible voltage mapping with minimal perturbation of the surrounding electric field. The precise positioning and insulation of the interconnecting traces address a key limitation of prior approaches, where stabilization of electrodes often required support structures that could distort the local fields.^15,16^ Moreover, the sensor-embedded TMM fabrication approach used here can be adapted to create anatomically accurate multilayer phantoms by modifying the molding technique, highlighting the scalability of the platform for future studies involving realistic tissue geometries. For example, MRI or CT scans can be used to segment tissue layers into ∼5 mm thick slabs with heterogeneous conductivities, which can then be 3D-printed as molds with planar surfaces suitable for sensor integration. By repeating this process for each layer and stacking them sequentially, we can reproduce complex volumetric tissue geometries, such as a realistic head, highlighting the scalability of the platform for future studies involving realistic tissue architectures.

More recently, emerging techniques such as carbon-based microelectrode arrays,^27–29^ printable conductive inks,^30^ and conducting polymer hydrogel sensors,^31^ have been explored to achieve micrometer-scale resolution in soft or biological media. Microelectrode arrays fabricated on continuous flexible substrates (e.g., PDMS or polyimide) offer excellent conformality, but the supporting sheets introduce additional dielectric interfaces that can perturb the field when multiple layers are stacked in volumetric phantoms. Printed conductive-ink electrodes (inkjet or screen-printed) enable rapid, large-area patterning and scalable fabrication, yet their conductivity is often nonuniform and orders of magnitude lower than bulk metals, reducing signal fidelity. Hydrogel-based sensors provide high mechanical compliance and can be molded into multilayer phantoms with complex geometry, but dehydration and time-dependent instability can limit the duration and reliability of measurements. In this context, the present platform emphasizes a balanced design that combines mechanically robust, highly conductive metal point sensors with electrical insulation and precisely defined spatial placement, minimizing field perturbation while remaining compatible with scalable multilayer phantom architectures.

## 3 Conclusion and Outlook

This study addresses a fundamental challenge in advancing precision electrical stimulation protocols: the accurate, high-resolution mapping of voltage distributions within tissue-mimicking materials (TMMs). Building on our earlier material-driven platform, we improved the sensor technology by engineering a novel insulated copper sensor architecture within conductive PDMS/MWCNT composites together with a robust, modular measurement interface. This combination enabled dense, reliable sensor networks that minimized field distortion and maximized the mapping accuracy. By directly comparing this new insulated design to a prior noninsulated platform, we demonstrated the critical role of sensor insulation and stable measurement interface design for achieving reliable and spatially resolved voltage measurements.

Our findings show that sensor insulation significantly reduces field distortion and enhances spatial resolution in voltage mapping, with insulated sensors suppressing unintended current pathways and producing more symmetric and sharper local voltage gradients beneath stimulation electrodes. In contrast, the noninsulated sensor platform exhibited lower overall voltage drops and significant asymmetry, attributable to current spreading and diffusing from exposed copper tracks. The stability and fidelity of these measurements were further supported by our integrated, multiplexer-based interface, which enables semiautomated differential data acquisition across up to 15 sensor nodes. The integrity of the sensor insulation was independently validated using saline bath tests, in which an insulated sensor immersed in high-conductivity saline (1.07 S/m) exhibited no detectable conductive current path under 1 mA and 1 kHz excitation, confirming effective electrical isolation; only the expected residual capacitive coupling through the dielectric layer was observed.

The TMM exhibits predominantly resistive behavior, supporting the quantitative interpretation of mapped voltage drops. Future work will include finite-element-method (FEM) simulations of the TMM, including the insulated sensors, to further validate the experimentally observed voltage distributions and quantify the effects of the sensors on local electric fields.

While the present study focuses on a homogeneous, single-layer TMM (conductivity ∼0.24 S/m), biological tissues comprise multiple layers with distinct and often anisotropic conductivities. In this work, a specific MWCNT concentration was chosen as an example to demonstrate the methodology; however, the conductivity value can be adjusted in future studies by tuning the MWCNT concentration to achieve different conductivity levels.^22^ With the stable and modular measurement interface developed in this work, the sensor-embedded platform can be extended in the future to investigate voltage distributions in nonhomogeneous, multilayered, and anisotropic tissue-mimicking materials. The influence of different stimulation frequencies should also be considered in such cases, as higher frequencies or lower TMM conductivity can reduce the relative dominance of the resistive path and could enhance sensor-insulation-induced capacitive effects.

The versatility of our sensor insulation technique paves the way for the targeted placement and study of individual voltage sensors—not only in simple volume conductor geometries but also in multilayer TMMs (e.g., arm phantoms for electromyostimulation) and anatomically complex models such as brain phantoms for neuromodulation, electroencephalography source localization, and electrical impedance tomography research.

## 4 Methods

### 4.1 TMM Fabrication

TMM for sensor platforms was prepared by dispersing 1.5 wt % MWCNTs (CAS 308068-56-6, Sigma-Aldrich) into 60 g of PDMS base (Elastosil RT 604 Part A, Wacker Chemie AG) using a magnetic stirrer (RCT Basic, IKA) for 1 h. Mixing was conducted in a temperature/humidity cabinet (LHU-113, Espec) at 23 ± 0.5 °C and relative humidity of 40 ± 2%. PDMS curing agent (Elastosil RT 604 Part B, Wacker Chemie AG) was then added at a 10:1 (A:B) mass ratio. Due to the high viscosity caused by the MWCNT content, Part B was loaded with a syringe, dispensing half at the bottom and half at the top of the mixture to promote homogeneity. The mixture was further stirred for 10 min and degassed using a vacuum desiccator (Model 5311, Kartell).

### 4.2 TMM Characterization

The TMM prepolymer was cast into a custom resin mold for four-wire measurement and cured in a muffle furnace (Model M104, Heraeus) at 70 °C for 2 h. Electrical resistivity was determined using a precision LCR meter (Model LM3536, Hioki) over a frequency sweep from 4 Hz to 1 kHz. The observed frequency-independent conductivity confirmed a predominantly resistive response and adequate MWCNT percolation within the PDMS matrix.

### 4.3 Laser Ablation of Copper Foil

Copper foil patterning for noninsulated and insulated sensor platforms was performed using a DPL Smart Marker II laser (ACI Laser, Germany) with a pulsed laser beam at a wavelength of 1064 nm. The electrode array design was prepared in CAD software and imported into laser machining software, which also enabled a bounding box feature for precise sample alignment within the laser chamber. Key laser ablation parameters for effective structuring of the 35 μm copper foil were as follows: laser power set to 50%, scan speed 50 mm/s, repetition frequency 5.6 Hz, pulse width 3 μs, and 23 beam passes. These settings consistently yielded well-defined copper sensor arrays within the TMM structure.

#### 4.3.1 Fabrication of Insulated Sensors

A step-by-step overview of the entire fabrication process is provided in Supporting Information Figure S6, which illustrates each major stage of sensor encapsulation, patterning, and integration described below. Insulated sensor arrays were fabricated by sequentially encapsulating copper foil (35 μm, Goettle Leiterplattentechnik GmbH) with PDMS (ADDV-25 Blue, R&G Faserverbundwerkstoffe GmbH), mixed 1:1 by weight. A custom 3D-printed resin mold (Rigid 4K, Formlabs) was employed with profiles designed to yield two uniform 0.5 mm thick insulation layers on either side of the copper foil. The copper foil was placed onto the flat mold base and temporarily fixed with kapton tape along the mold edge for alignment. Degassed PDMS was applied via doctor blading to produce a uniform first insulation layer and then thermally cured at 65 °C for 20 min in an oven (T6060, Heraeus, Thermo Fisher Scientific).

After initial curing, the assembly was flipped to expose the opposite side of the foil. The sensor array pattern was imported from CAD sketches using the manufacturer’s software (V2, Magic Mark) and defined via laser ablation using the optimized parameters described above. To facilitate a careful removal of unwanted copper foil without exerting excessive pulling force—which could cause delamination from the PDMS surface—multiple grid lines were incorporated into the layout. These segmented the copper pattern, allowing precise extraction of foil segments with fine-tipped tweezers while preserving the integrity of the sensor and insulation layers.

A second 0.5 mm PDMS insulation layer was applied and cured as described above, encapsulating the structured foil. The insulation pattern was then defined by additional laser ablation and manual removal of excess PDMS, guided by laser-aligned reference features. For defining the insulation layout, the CAD design for the insulation pattern (see Supporting Information Figure S3) was imported into the laser device. Laser ablation parameters for PDMS were optimized through preliminary testing: power 75%, speed 35 mm/s, repetition frequency 2 Hz, and pulse width of 3 μs. The cutting depth was controlled by adjusting the number of beam passes: five passes were used for shallow cuts over sensing node regions (facilitating later manual removal of insulation from one side of each node), while 12 passes were applied to fully ablate PDMS in designated regions and delineate the final insulation geometry. After laser processing, unwanted PDMS segments were precisely removed by using fine-tipped tweezers.

### 4.4 Stable Measurement Interface

The voltage mapping system was built as a modular platform comprising a sensor interface board and a signal processing and control unit (Figure 3b).

The sensor interface board was fabricated as a printed circuit board (PCB) (Fusion 360, Autodesk), designed with a central cavity to accommodate the TMM and to facilitate a robust electrical connection to each embedded sensor via presoldered wires and female pin-headers. Signal breakout was routed through dual FFC connectors for a flexible connection to downstream electronics. For applications involving multilayer phantoms, identical adapter boards can be stacked, providing independent access to sensor nodes in each layer.

Signals from the sensor adapter were routed to multiplexer boards (MUX36S16, Texas Instruments), configured for 16:1 channel selection, and controlled digitally through a microcontroller unit (STM32 Nucleo-64, STMicroelectronics) via GPIO pins. Two multiplexers enabled flexible differential measurement protocols, supporting an arbitrary selection of sensor pairs from the same layer.

The selected signal was amplified using an instrumentation amplifier (AD620A, Analog Devices) with three selectable gain settings, providing a high input impedance of 10 GΩ and substantial common-mode noise rejection. Downstream analog filtering included a modular band-pass filter (0.1 Hz-1 kHz) and a passive twin-T notch filter (50 Hz) to minimize AC-induced measurement noise. Filtered signals could be acquired in real-time using an oscilloscope (RTC1002, Rhode & Schwarz), with a sampling rate of 980 kSa per second.

The system was powered by a dedicated dual-rail (±18 V analog, 5 V digital) supply, regulated via a two-stage power management circuit employing a buck converter (LM2596, Texas Instruments), followed by a linear regulator (LM1084, Texas Instruments) to reduce voltage ripple for the digital components. This architecture ensured robust, low-noise voltage mapping, the rapid switching of measurement channels, and straightforward integration with complex sensors.

### 4.5 Voltage Mapping within TMMs

Differential voltage signals were recorded directly in the time domain using an oscilloscope, and RMS values were subsequently calculated offline for analysis. Sensor 1 was chosen as the reference sensor in each TMM platform for differential voltage measurements, as it is located at the maximum distance from the stimulation axis while remaining on the same plane. Table 1 shows the key experimental parameters of the insulated and noninsulated TMM platforms.

**Table 1:** Summary of Key Experimental Parameters Used in TMM Platforms

parameter	value
TMM material	carbon nanotubes (1.2 wt %) doped in PDMS
sensor material	copper −35 μm thick
stimulation electrode material	platinum/iridium (commercial neurostimulation electrodes)
insulation material	polydimethylsiloxane (PDMS)
mode of stimulation	biphasic, sinusoidal alternating current
stimulation waveform	14 μA at 100 Hz
TMM dimensions	7 cm (length), 7 cm (width), 0.5 cm (thickness)
TMM conductivity	0.24 S/m
sensor track dimensions	1 to 2 cm (length), 0.3 mm (width)
sensor pitch	1 cm
sensing element diameter	1.6 mm
sensor insulation dimensions	1 to 2 cm (length—depending on the track length), 1 mm (width), 1 mm (thickness)

## Supplementary Material



## Notes

Photograph of the sensor array after laser ablation and soldering of connecting wires; schematic of the custom-designed 3D-printed resin mold; CAD sketch of the insulation pattern; schematic of the experimental setup and insulation integrity testing results; optical profilometry analysis of insulation layer thickness; fabrication process schematics of insulated sensors; impedance analysis of insulated sensors; RMS voltage heatmaps without interpolation; FEM simulation results; raw input and output voltage signals; and differential RMS voltage distributions in other insulated and noninsulated platforms (PDF)

## References

[1] Chen C., Bai X., Ding Y., Lee I.-S. (2019). Electrical Stimulation as a Novel
Tool for Regulating Cell Behavior in Tissue Engineering. Biomater. Res..

[2] Poompavai S., Gowri Sree V. (2018). Dielectric
Property Measurement of Breast—Tumor
Phantom Model Under Pulsed Electric Field Treatment. IEEE Trans. Radiat. Plasma Med. Sci..

[3] Heikenfeld J., Jajack A., Rogers J., Gutruf P., Tian L., Pan T., Li R., Khine M., Kim J., Wang J., Kim J. (2018). Wearable Sensors:
Modalities, Challenges, and Prospects. Lab Chip.

[4] Lipp C., Laamari L., Bertsch A., Podlesek D., Bensafi M., Hummel T., Brugger J. (2025). Devices for
the Electrical Stimulation
of the Olfactory System: A Review. Biosens.
Bioelectron..

[5] Edwards C. A., Kouzani A., Lee K. H., Ross E. K. (2017). Neurostimulation
Devices for the Treatment of Neurologic Disorders. Mayo Clin. Proc..

[6] Karamian B. A., Siegel N., Nourie B., Serruya M. D., Heary R. F., Harrop J. S., Vaccaro A. R. (2022). The Role of Electrical
Stimulation
for Rehabilitation and Regeneration after Spinal Cord Injury. J. Orthop. Traumatol..

[7] Pérez C., Leite J., Carvalho S., Fregni F. (2016). Transcranial Electrical
Stimulation (tES) for the Treatment of Neuropsychiatric Disorders
Across Lifespan. Eur. Psychol..

[8] Moreno-Duarte I., Morse L. R., Alam M., Bikson M., Zafonte R., Fregni F. (2014). Targeted Therapies Using Electrical
and Magnetic Neural
Stimulation for the Treatment of Chronic Pain in Spinal Cord Injury. NeuroImage.

[9] Uhrhan K., Schwindt E., Witte H. (2024). Fabrication
and Dielectric Validation
of an Arm Phantom for Electromyostimulation. Bioengineering.

[10] Agboada D., Vicario C. M., Wischnewski M. (2025). Editorial:
Transcranial Electrical
Stimulation (tACS, tDCS, tRNS) in Basic and Clinical Neuroscience:
Current Progress and Future Directions. Front.
Hum. Neurosci..

[11] Liu A., Vöröslakos M., Kronberg G., Henin S., Krause M. R., Huang Y., Opitz A., Mehta A., Pack C. C., Krekelberg B., Berényi A., Parra L. C., Melloni L., Devinsky O., Buzsáki G. (2018). Immediate
Neurophysiological Effects of Transcranial Electrical Stimulation. Nat. Commun..

[12] Bland N. S., Sale M. V. (2019). Current Challenges:
The Ups and Downs of tACS. Exp. Brain Res..

[13] Bikson M., Rahman A., Datta A. (2012). Computational
Models of Transcranial
Direct Current Stimulation. Clin EEG Neurosci.

[14] Kim D., Jeong J., Jeong S., Kim S., Jun S. C., Chung E. (2015). Validation of Computational Studies
for Electrical Brain Stimulation
With Phantom Head Experiments. Brain Stimul..

[15] Hunold, A. Transcranial Electric Stimulation: Modeling, Application, Verification, 2021. DOI: 10.22032/dbt.49291.

[16] Morales-Quezada L., El-Hagrassy M. M., Costa B., McKinley R. A., Lv P., Fregni F. (2019). Transcranial
Direct Current Stimulation Optimization
- From Physics-Based Computer Simulations to High-Fidelity Head Phantom
Fabrication and Measurements. Front. Hum. Neurosci..

[17] Jung Y.-J., Kim J.-H., Kim D., Im C.-H. (2013). An Image-Guided
Transcranial Direct Current Stimulation System: A Pilot Phantom Study. Physiol Meas.

[18] Hunold A., Machts R., Haueisen J. (2020). Head Phantoms for Bioelectromagnetic
Applications: A Material Study. Biomed Eng.
Online.

[19] Jain, K. C. ; Goldschmidtboeing, F. ; Hügel, P. ; Srivastava, R. ; Dümpelmann, M. ; Woias, P. ; Comella, L. M. Brain Phantom with Embedded Voltage Sensors for Transcranial Electrical Stimulation. TechRxiv.23972359/v1, 10.36227/techrxiv.176186872.23972359/v1.

[20] Jain, K. C. ; Helm, J.-H. ; Cayoglu, M. D. ; Goldschmidtboeing, F. ; Jerg, K. ; Hesser, J. ; Woias, P. ; Comella, L. M. Sensor-Embedded Tissue-Mimicking Material for Long-Term Transcranial Electrical Stimulation. In 2025 10th International Conference on Intelligent Informatics and Biomedical Sciences (ICIIBMS); IEEE: Okinawa, Japan, 2025, 2025; pp 400–405.

[21] AD620 Datasheet and Product Info | Analog Devices. https://www.analog.com/en/products/ad620.html (accessed Jan, 15 2026).

[22] Jain, K. C. Tissue-Mimicking Material for Long-Term Transcranial Electrical Stimulation. In 2024 16th Biomedical Engineering International Conference (BMEiCON); IEEE: Pattaya, Thailand, 2024.

[23] Bensalah F., Pézard J., Haddour N., Erouel M., Buret F., Khirouni K. (2021). Carbon Nano-Fiber/PDMS Composite Used as Corrosion-Resistant
Coating for Copper Anodes in Microbial Fuel Cells. Nanomaterials.

[24] Kim Y.-H., Jeong M.-G., Seo H. O., Park S.-Y., Jeong I.-B., Kim K.-D., Cho S. M., Lim D. C., Kim Y. D. (2012). Preparation
of Ultrathin Polydimethylsiloxane-Coating on Cu as Oxidation-Protection
Layer. Appl. Surf. Sci..

[25] Magsood H., Hadimani R. L. (2021). Development of Anatomically
Accurate Brain Phantom
for Experimental Validation of Stimulation Strengths during TMS. Mater. Sci. Eng. C Mater. Biol. Appl..

[26] Magsood, H. ; Serrate, C. H. A. ; El-Gendy, A. A. ; Hadimani, R. L. Anatomically Accurate Brain Phantoms and Methods for Making and Using the Same. US11373552B2, June 28, 2022. https://patents.google.com/patent/US11373552B2/en (accessed Jan, 01 2026).

[27] Adly N., Weidlich S., Seyock S., Brings F., Yakushenko A., Offenhäusser A., Wolfrum B. (2018). Printed Microelectrode Arrays on
Soft Materials: From PDMS to Hydrogels. npj
Flex Electron.

[28] Meacham K. W., Giuly R. J., Guo L., Hochman S., DeWeerth S. P. (2008). A Lithographically-Patterned,
Elastic Multi-Electrode Array for Surface Stimulation of the Spinal
Cord. Biomed. Microdevices.

[29] Byun I., Coleman A. W., Kim B. (2013). Transfer of
Thin Au Films to Polydimethylsiloxane
(PDMS) with Reliable Bonding Using (3-Mercaptopropyl)­Trimethoxysilane
(MPTMS) as a Molecular Adhesive. J. Micromech.
Microeng..

[30] Veerapandian S., Kim W., Kim J., Jo Y., Jung S., Jeong U. (2022). Printable
Inks and Deformable Electronic Array Devices. Nanoscale Horiz..

[31] Gamboa J., Paulo-Mirasol S., Estrany F., Torras J. (2023). Recent Progress in
Biomedical Sensors Based on Conducting Polymer Hydrogels. ACS Appl. Bio Mater..

