# Supplementary material for: Integrated Sensor-Composite Material Platform for High-Resolution Voltage Mapping in Tissue-Mimicking Models
Source: ACS Omega. 2026 Mar 16;11(12):19271–81. doi: 10.1021/acsomega.5c12602 (PMC13044841; doi:10.1021/acsomega.5c12602)
Supplement: Supplementary file 1 [file ao5c12602_si_001.pdf]

Integrated Sensor-Composite Materials Platform for High-Resolution Voltage Mapping in Tissue-Mimicking Models

Kajal C. Jain[*1], *Richa Srivastava*[1], *Armin Jamali*[1,2], *Frank Goldschmidtboeing*[1,2], *Peter Woias*[1,2], *Laura M. Comella*[2,3]

[1] Laboratory for Design of Microsystems, Department of Microsystems Engineering - IMTEK, University of Freiburg, Freiburg im Breisgau, 79110, Germany

[2] Cluster of Excellence livMatS, FIT - Freiburg Center for Interactive Materials and Bioinspired Technologies, University of Freiburg, Freiburg im Breisgau, 79110, Germany

[3] Institute of Energy Efficient Mobility, Karlsruhe University of Applied Sciences, Karlsruhe, 76133, Germany

Corresponding Author

*E-Mail: kajal.jain@email.uni-freiburg.de

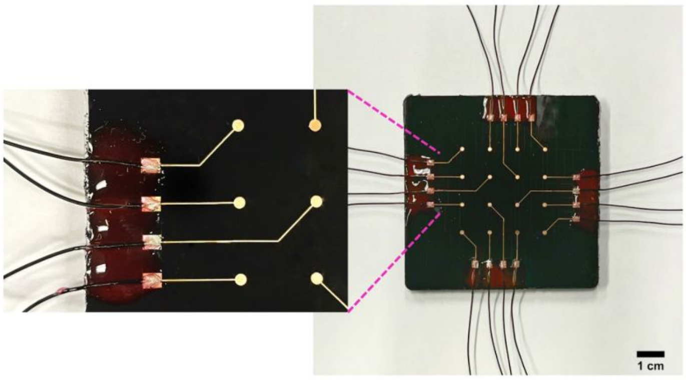

Figure S1. Photograph of the sensor array after laser ablation and soldering of connecting wires to the exposed solder pads. The connecting wires were fixated with addition-cure silicone (ADDV-42 (Red), R&G Faserverbundwerkstoffe GmbH) to protect the copper sensors from mechanical stress during measurements.

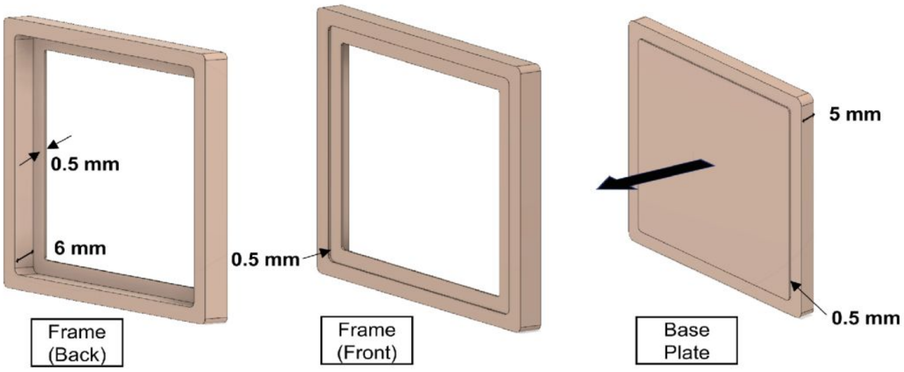

Figure S2. Schematic of the custom-designed 3D-printed resin mold used for fabrication of the insulated sensor platform. The mold features a 5 mm deep cavity for the main TMM body and two separate 0.5 mm profiles that together define the 1 mm-thick insulation layer.

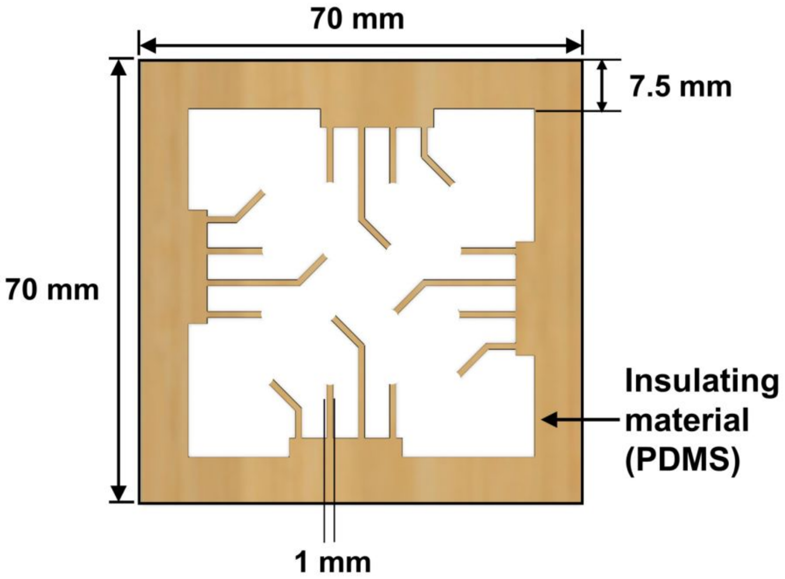

Figure S3. CAD sketch of the insulation pattern used for laser cutting. The design was imported into the laser cutting device to define the regions for ablation through the 1 mm-thick PDMS insulation layer around the copper tracks and solder pads.

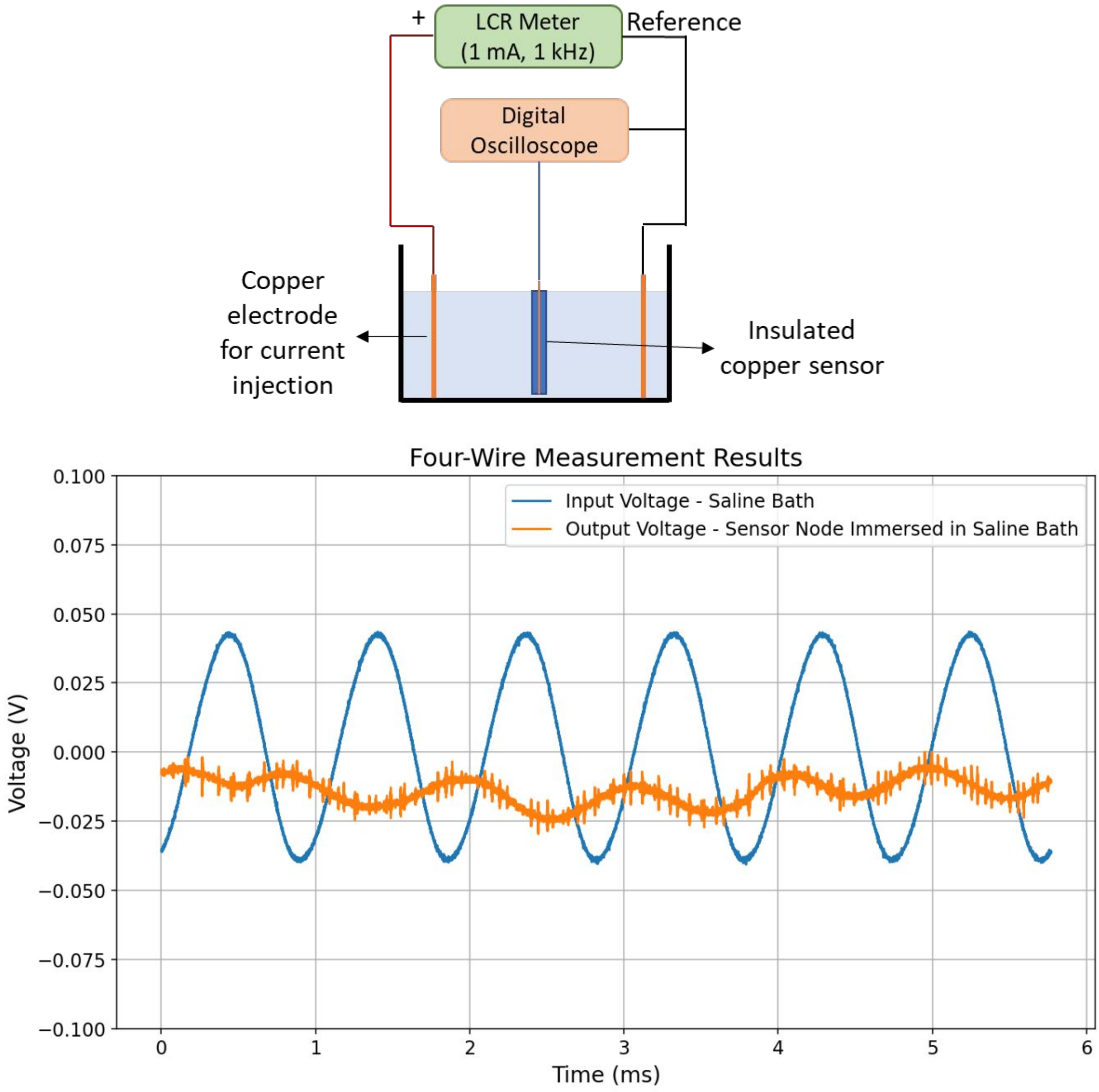

Figure S4. Schematic of the experimental setup (top) and results (bottom) for insulation integrity testing of the encapsulated copper sensor. The schematic shows an insulated sensor immersed in a saline bath (conductivity 1.07 S/m), with uninsulated solder pads and connecting wires positioned outside the solution. A constant current of 1 mA at 1 kHz was applied, and no conductive pathway was detected, confirming effective electrical isolation. Residual capacitive coupling between the sensor and the saline was observed, as expected due to the dielectric insulation layer.

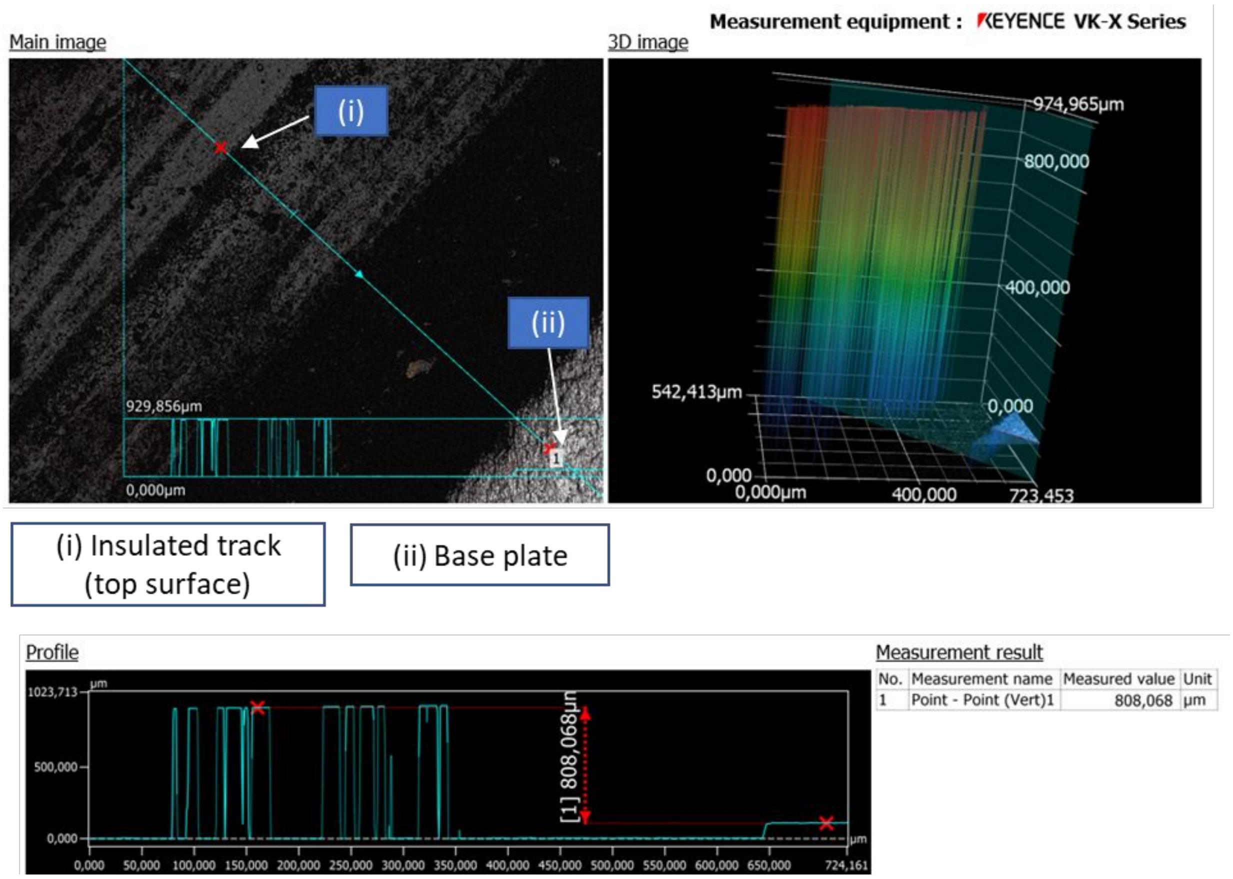

Figure S5. Optical profilometry analysis of the insulation layer thickness of a single track: Main image – Reflective image of the sample with marked focal point located on (i) top surface of insulated track, and (ii) the base plate on which the insulated tracks are placed. The scan line joining (i) and (ii) along which the profilometry scan was performed;

3D image – Reconstructed three-dimensional profile of the insulation region, with multiple discrete scan lines highlighting the surface topography.

Profile graph – Cross-sectional line scan showing lateral distance and measured height (Z axis), with the measured thickness between the two marked focal points shows that the insulation thickness is 808.07 µm. The presence of valleys along the insulation profile, rather than a perfectly straight edge, is indicative of optical interference resulting from the underlying embedded copper tracks. The flat zero-line between the end of the insulation surface and the beginning of the base plate indicates the absence of a detectable surface due to loss of optical focus in that region, likely caused by the V-shaped/sloped edge geometry resulting from the laser cutting process. This flat region extends over ~300,000 µm along the lateral axis, which is consistent across all three sensor tracks that were analyzed.

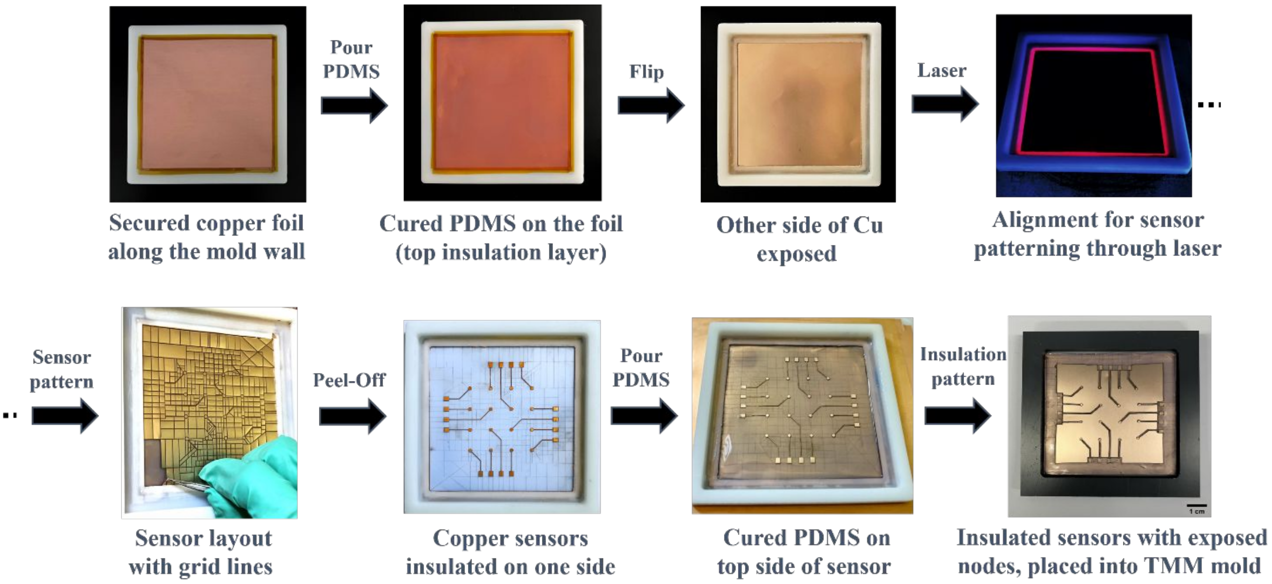

Figure S6. Step-by-step schematic of the fabrication process for the insulated sensor platform. The figure illustrates each major stage, including copper foil placement, sequential PDMS insulation layer application and curing, laser ablation for sensor and insulation patterning, removal of excess copper and PDMS, exposure of sensing nodes, and placement into the TMM mold for material integration.

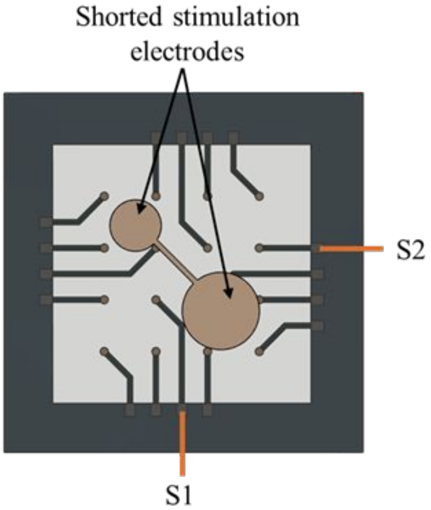

(a) Insulated TMM platform (Top view)

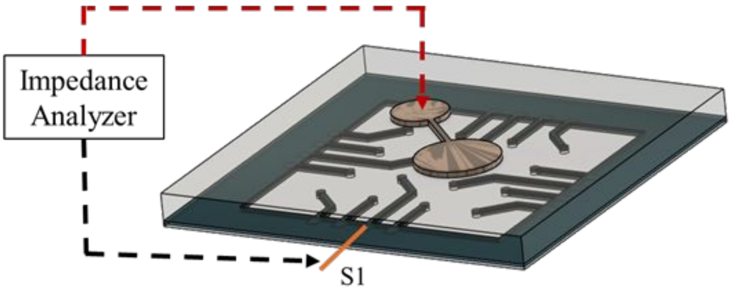

(b) Experimental setup

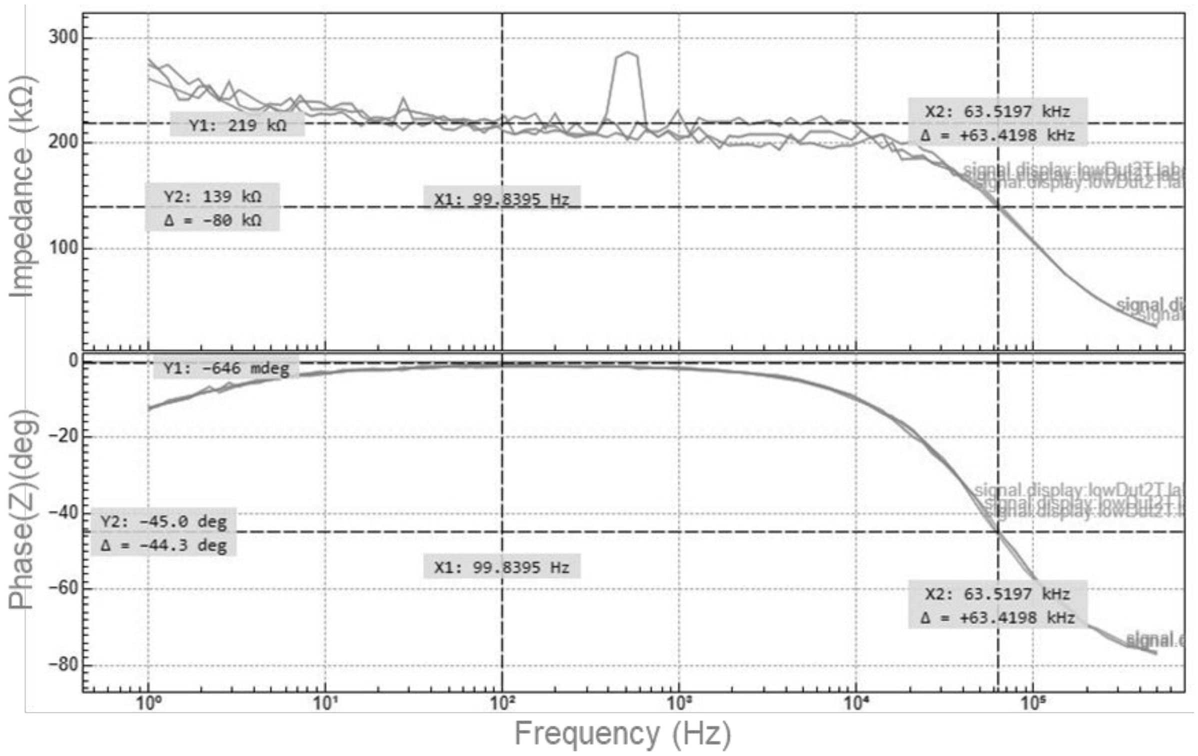

(c) Impedance and Phase Measurements – Sensor S1

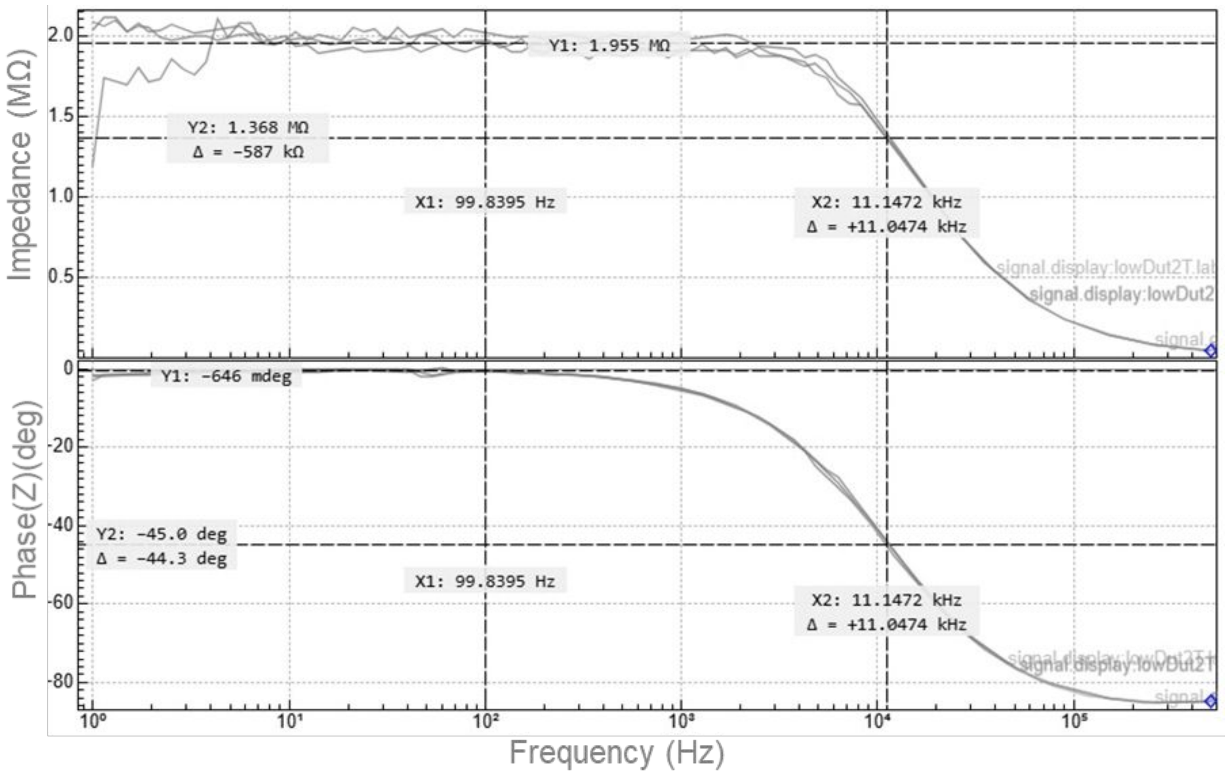

(d) Impedance and Phase Measurements – Sensor S2

Figure S7. Impedance analysis of Sensors S1 and S2 in the insulated TMM platform over a frequency range of 1 Hz to 500 kHz. The stimulation electrodes were electrically shorted, and electrical contact with the top surface of the TMM was established using a thin layer of conductive paste to replicate the stimulation boundary conditions used in voltage-mapping experiments. Sensor S1 was selected due to its maximum track length and proximity to the stimulation site, while Sensor S2 was selected due to its shortest track length and location farther from the stimulation site.

(a) Relative positions of the stimulation electrodes (top surface of the TMM) and Sensors S1 and S2 (embedded at the bottom of the TMM);

(b) Experimental setup showing the measurement terminals of the impedance analyzer (MFIA 500 kHz / 5 MHz Impedance Analyzer, Zurich Instruments AG, Switzerland);

(c-d) Measured impedance magnitude and phase response for Sensors S1 and S2, respectively. At 100 Hz, the phase angle remains close to 0° for both sensors (marked by X1), indicating that the impedance is dominated by resistive contributions under the measurement conditions. Therefore, capacitive coupling through the sensor insulation is negligible at the operating frequency used for voltage-mapping experiments.

A noticeable phase shift is observed at low frequencies (approximately 1–10 Hz) for Sensor S1, whereas no comparable low-frequency phase shift is observed for Sensor S2. Similar low-frequency phase behavior is also observed in other sensors located closer to the stimulation site within the same TMM sample, indicating that this behavior is spatially correlated with proximity to the stimulation electrodes rather than being sensor-specific. This low-frequency capacitive behavior is attributed to electrode polarization at the stimulation electrode–TMM interface, and is therefore expected to be more pronounced for sensors located closer to the stimulation site.

At higher frequencies, a transition toward capacitive-dominated behavior (phase of - 45°) is observed for both sensors, albeit at different frequencies. For Sensor S1, a pronounced phase shift indicative of capacitive dominance emerges at frequencies above approximately 63 kHz, whereas for Sensor S2, a similar transition occurs at lower frequencies, above approximately 11 kHz. This sensor-dependent high-frequency behavior suggests that the TMM–sensor system exhibits a frequency-dependent attenuation characteristic consistent with low-pass filtering, with effective cutoff frequencies that vary across sensors. A detailed investigation of these effects is beyond the scope of the present study and will be addressed in future work.

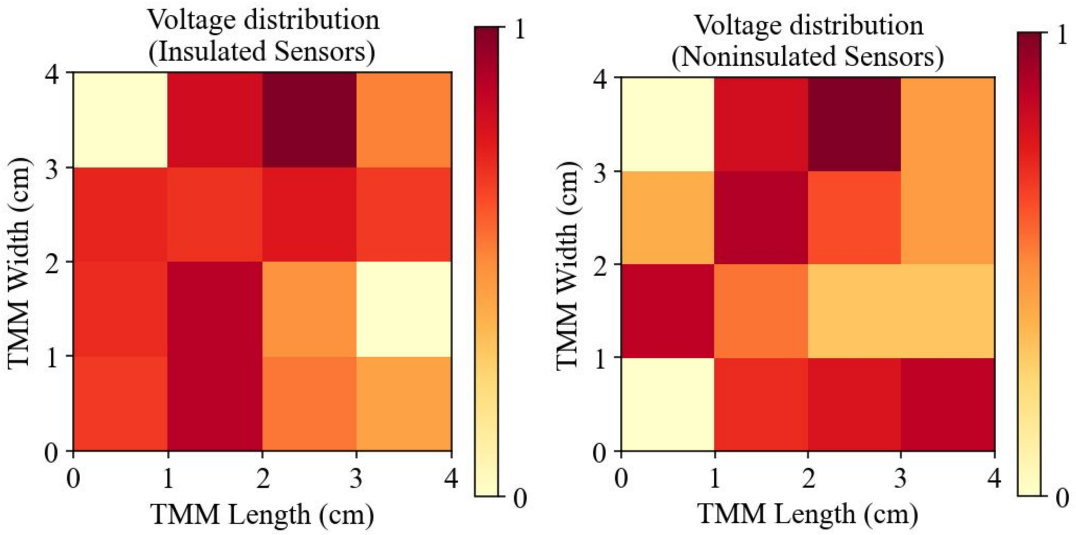

Figure S8. Heatmaps of normalized RMS voltage distributions across the TMM surface without interpolation. Each 1 × 1 cm grid cell represents the voltage measured at a single measurement node with an effective sensing diameter of approximately 1.6 mm, located at the center of the grid cell. In the normalized scale, the minimum-maximum voltage values corresponding to 0 and 1 are 0.357 V – 0.391 V (for insulated sensors) and 0.455 V – 0.471 V (for non-insulated sensors).

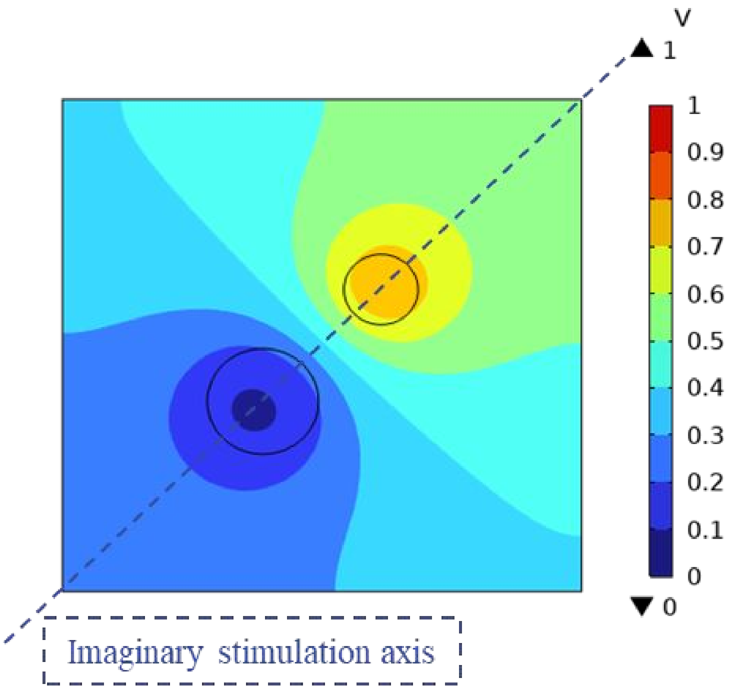

Figure S9. Finite-element-method (FEM) simulation of the TMM performed in COMSOL Multiphysics 6.2, showing the expected voltage distribution under electrical stimulation. A symmetric field distribution is observed along the stimulation axis, with the highest field directly beneath the electrodes and gradually decreasing away from them. These contours qualitatively match the measured voltage patterns in the TMM. However, insulated sensors should be included in the FEM model in future to allow quantification of the local field disturbances caused by the sensors.

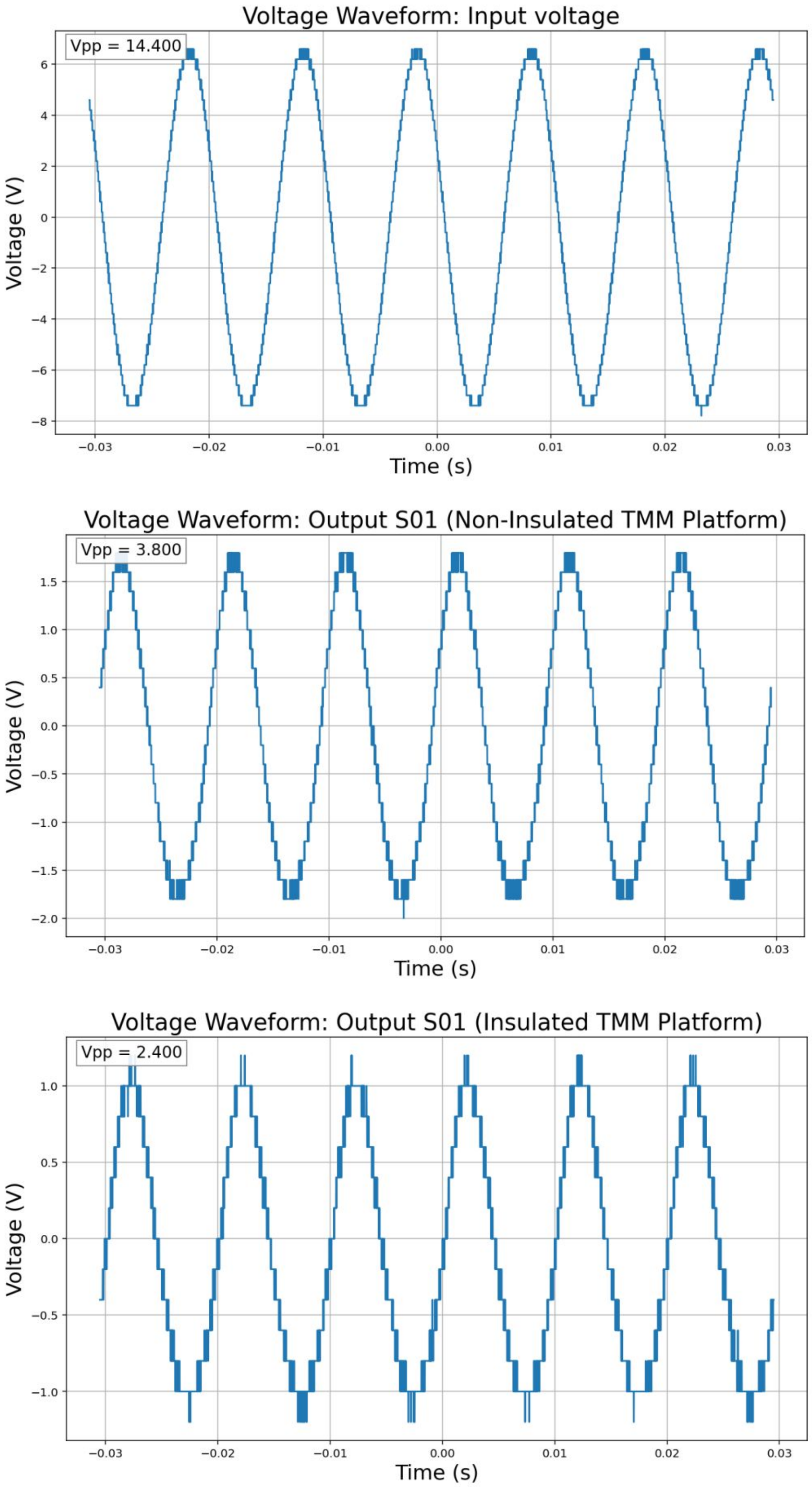

Figure S10. Figures showing raw input voltage signal applied to the TMM platforms at 100 Hz, and the raw output voltage signals recorded (before filtering) from the non-insulated and the insulated sensor platform.

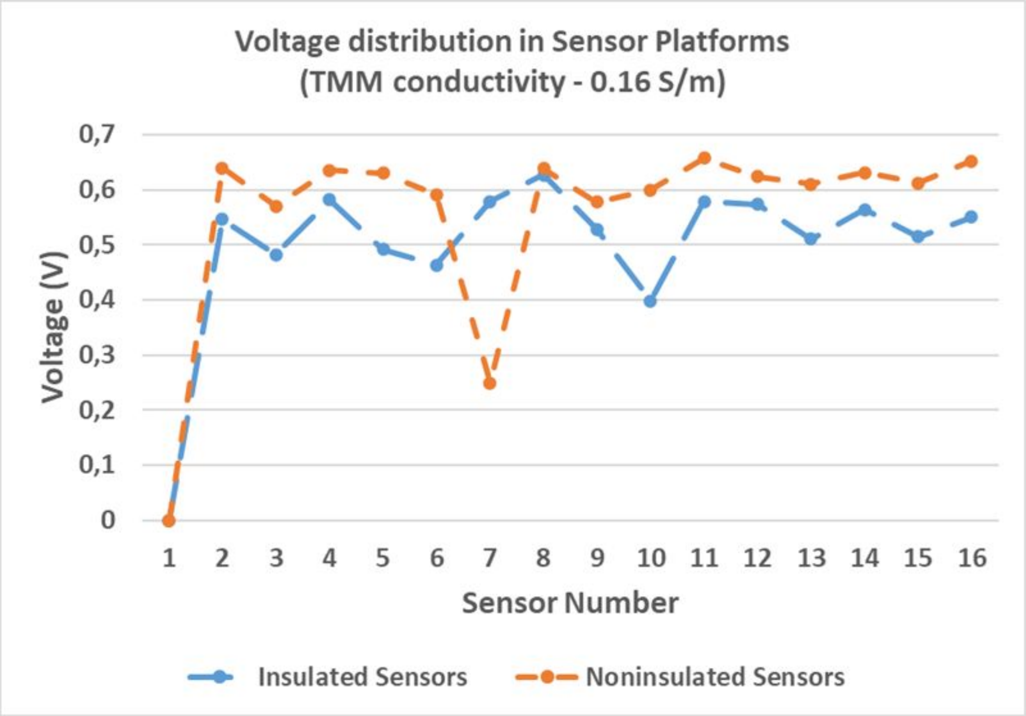

Figure S11. Differential root-mean-square (RMS) voltage distributions for insulated and non-insulated TMM platforms under identical AC stimulation conditions (14.4 Vpp, 100 Hz, position of stimulation electrodes). Absolute voltages were measured with respect to a common system ground of the measurement setup, and differential voltages were calculated with respect to Sensor 1. The TMMs were prepared by doping 1.2 wt% MWCNTs in PDMS, yielding a conductivity of 0.16 S/m. The voltage distribution pattern in distinct TMMs under different stimulation and measurement conditions show similar pattern observed in Figure 4b. The Pearson correlation coefficient between the insulated and non-insulated platforms was 0.76 across all sensors, and 0.95 after excluding one sensor in the non-insulated platform with poor contact (Sensor 7), highlighting the strong preservation of spatial voltage patterns. The non-insulated TMM platform exhibits more homogeneous voltage distribution compared to the insulated platform, indicating higher spatial resolution of the insulated sensors. Symmetric distribution is observed across Sensor 8 in the insulated TMM platform, as expected in a homogeneous TMM layer. The sharp voltage drop at Sensor 7 of the non-insulated platform can be attributed to poor contact with the TMM, as the non-insulated sensors are prone to delamination (also observed in Figure 4b, Sensor 16 of non-insulated TMM platform).
